# Portal of Juglandaceae: A comprehensive platform for Juglandaceae study

**DOI:** 10.1038/s41438-020-0256-x

**Published:** 2020-03-15

**Authors:** Wenlei Guo, Junhao Chen, Jian Li, Jianqin Huang, Zhengjia Wang, Kean-Jin Lim

**Affiliations:** State Key Laboratory of Subtropical Silviculture, Zhejiang A&F University, Lin’an District, 311300 Hangzhou, Zhejiang China

**Keywords:** Bioinformatics, Systems analysis

## Abstract

Juglandaceae species are plants of great economic value and have been cultivated, domesticated, and utilized by human society for a long time. Their edible, nutrient-rich nuts and tough, durable wood have attracted the attention of botanists and breeders. With the advent of the genomics era, genome sequencing of the Juglandaceae family has been greatly accelerated, and a large amount of data has been generated. In this paper, we introduce the Portal of Juglandaceae (PJU), a tool to bring all these data together. The PJU contains genomes, gene-coding sequences, protein sequences, various types of annotation information, expression data, and miRNA data, which are configured with BLAST, JBrowse, and our self-developed synteny analysis tool. The PJU has a user-friendly and straightforward interface that performs a variety of query tasks with a few simple operations. In the future, we hope that the PJU will serve as a hub for the study of the Juglandaceae family.

## Introduction

The Juglandaceae family, with eight genera and more than 60 species, has a wide distribution in North America, Europe, and Asia^1^. Throughout the history of human society, Juglandaceae species have been cultivated, bred, and harvested^2^. The two most well-known genera of Juglandaceae are *Carya* and *Juglans*. Most species in these two genera not only have edible, delicious, and highly nutritious nuts but also produce durable and decay-resistant wood^3^. For example, the timber of *J. nigra* (black walnut) is highly recognized as the highest grade hardwood in the western world^4^. The wood is characterized by its beautiful grain, dark color, high stability, and moderate density^4^.

Another highly valued Juglandaceae family commodity is nuts. The common walnut (*J. regia*), pecans (*C. illinoinensis*), and Chinese hickory (*C. cathayensis*) are the most consumed commercial nuts from the Juglandaceae family^2^. Compared to other commercial nuts, pecans and Chinese hickory contain high contents of dietary fiber, minerals, and vitamins^5^. The properties of a variety of antioxidants in these nuts (e.g., *β*-carotene) make them sought after in the global nut market^6,7^.

Although the Juglandaceae family occupies a very significant position in the economic landscape, there are still several major biological questions about this family that need to be addressed: What are the molecular mechanisms underlying the high oil content of Juglandaceae nuts? What is the genetic background of differences among individuals in the same species? Such questions could have profound commercial implications. For instance, due to the naked terminal buds of Chinese hickory and its specific climate needs, its cultivation range is geographically restricted^8,9^. Breeding stress-tolerant and vigorous Juglandaceae tree varieties is a long-term objective in the research community. Utilizing genomic data to understand the mechanisms of plant development and stress responses could help in breeding.

Recently, various genomic databases have been constructed due to the rapid development of plant genome sequencing^10,11^. However, to date, no specific database is available for walnut research. Many genomic data about the Juglandaceae family^12–14^ are accessible in public databases, such as GigaDB^15^ and NCBI^16^; however, most of these genomic data are unprocessed and decentralized. The data are neither functionally annotated and clustered in gene families nor preformatted for searching. Scientists have to process the data themselves after downloading them from public databases. However, processing these data is a challenge, especially for those without a bioinformatics background. Hence, these databases are among the least useful tools for the scientific community.

As mentioned above, we believe it is necessary to establish a shared resource center that integrates genomic data and germplasm (phenotype) resources of the Juglandaceae family. We built a comprehensive database for the Juglandaceae family, “Portal of Juglandaceae” (PJU, www.juglandaceae.net), that integrates their genomes, transcriptomes, and phenotypic data. We then mined, analyzed, and clustered these data appropriately to allow researchers to utilize the data more efficiently. Most importantly, the PJU has a user-friendly web interface for the scientific community. The PJU is also integrated with numerous practical bioinformatics tools that help researchers and users search, browse, or retrieve desired information from the portal. We hope, in time, that the PJU will become a comprehensive genomic data platform for the scientific community, especially for the study of Juglandaceae.

## Construction of the PJU

### Acquisition of genomic and transcriptomic data

Our colleagues previously reported the reference genomes of Chinese hickory (*C. cathayensis*) and pecan (*C. illinoinensis*)^17^. Reference genome, general feature format (GFF), coding sequence (CDS), protein sequence (PEP), and RNA expression data sets were obtained from the supporting data in the GigaScience database^15^.

The reference genomes of six *Juglans* and one *Pterocarya* species (*J. cathayensi, J. hindsii, J. microcarpa, J. nigra, J. regia, J. sigillata, P. stenoptera*) in the PJU were obtained from a previous study under Bioproject PRJNA445704^18^. The types of genomic data mentioned above were also downloaded from NCBI. A summary of genomic data currently available in the PJU is presented in Table 1.

**Table 1:** Summary of the genome assembly statistics of 9 Juglandaceae taxa

Species	Estimated genome size (Mb)	Total assembly (Mb)	Number of scaffolds	N50 scaffold length (Mb)	Repeat sequence (Mb/%)
*Carya cathayensis*	721.33	706.43	5449	1.22	381.01/53.67
*Carya illinoinensis*	649.75	651.31	3860	1.08	334.55/50.43
*Juglans cathayensis*	582	797.89	332,634	0.145	325/23
*Juglans hindsii*	577	643.32	273,094	0.47	170/12
*Juglans nigra*	583	640.9	232,579	0.245	148/10
*Juglans regia*	571	651.68	186,636	0.64	299/21
*Juglans sigillata*	594	668.76	282,224	0.2	158/11
*Juglans microcarpa*	571	941.87	329,873	0.14	540/38
*Pterocarya stenoptera*	600	991.97	396,056	0.15	580/40

The transcriptome expression data of the Juglandaceae family were recalculated from fragments per kilobase of exon model per million mapped fragments (FPKM) to transcripts per million (TPM) to present the relative transcript expression levels appropriately^19^.

### Acquisition of phenotypic and physiological data

Germplasm resources are formed through thousands of years of natural evolution under different ecological conditions. They contain various potential genes and are important foundations for plant systematics research. Our group has been studying pecan germplasms from China and the United States for years. We have investigated essential features, such as flower phenology, fruit morphological indexes, fatty acids, and amino acid composition, in our pecan germplasm resources center. All the above data sets were converted to visual charts and are presented in the PJU.

### Gene annotation, family, and synteny search

In attempts to decipher the unknown functions of tens of thousands of protein CDSs in individual genomes, researchers often need to perform comparisons using a variety of databases to make inferences on the sequences’ possible functions. InterProScan^20^ was used (with local mode and full analysis parameters) to analyze the protein domain functions of Juglandaceae family genes using searches against the InterPro and Pfam databases^21^. The predicted relevant domain and site results were classified according to HMM models using default parameters. Each gene was annotated with Gene Ontology (GO)^22^ terms and the Kyoto Encyclopedia of Genes and Genomes (KEGG)^23^ to understand its function and location, respectively, in biochemical pathways.

The transcription factor families of Juglandaceae species were identified by searching against the Plant Transcription Factor Database^24^ using HMM models from the Pfam database (version 31.0)^21^. The HMMbuilder in the HMMER toolkit^25^ was also used (with default parameters) to assist in the identification process when an HMM model was not available.

Because of the close phylogenetic relationships among Juglandaceae species, there may be many homologous gene blocks in their genomes^26^. The Python version of MCScan^27^ (https://github.com/tanghaibao/jcvi/wiki/MCscan-(Python-version)) was used to find homologous gene blocks in genomes of the Juglandaceae family. The CDSs and coordinate files of nine species were extracted, and then the syntenic gene blocks between multiple genomes were obtained using the jcvi.compara module of Mcscan with the minspan parameter set at 30 genes.

### BLAST and JBrowse modules, flanking sequence finder

BLAST^28^, one of the most popular bioinformatics tools for sequence similarity searches, was installed in the PJU. This module supports FASTA file dragging and multiple searches against preformatted genomes. JBrowse^29^, built using JavaScript and HTML5, was embedded in the PJU to browse and visualize the desired genomes. In this module, smooth scrolling and scaling visualization from the genome to nucleotide base level is supported.

With the two tools described above, researchers can easily find homologous genes Juglandaceae species. To find promoters, we utilized GFF and genome data and developed the Flanking Sequence Finder, which can help researchers find the flanking sequence of a specific gene and the exon and intron sequences.

### Data integration and website construction

**Fig. 1:**
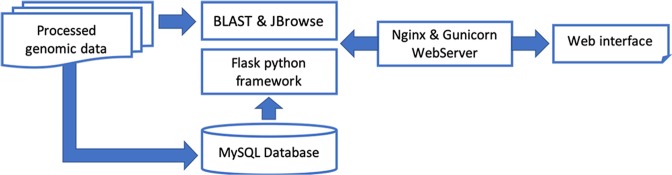
Overview of the Portal of Juglandaceae overall architecture. In short, processed genomic data are stored in a MySQL database on the server. A Flask framework-based website was built for visitors to utilize and download our data

We created and deployed a user-friendly website for the PJU to make it easy for the scientific community to use the platform. The web pages are set up on the Huawei cloud server, one of the safest and most stable cloud service providers in the world. The server runs on the Ubuntu Linux system, uses Nginx as a web server and is also a protector of the PJU web application. The PJU data sets are stored in a MySQL database. To date, nine genomes, 488,299 PEPs, 1,792,549 GO annotations, and 750 miRNAs (Table 2) have been downloaded, analyzed, and organized in the MySQL database. We used Flask (a Python microframework) to fit the database web application into a single Python project. The PJU utilizes the Python program to handle data analysis and interactive tasks. The PJU web pages are built using HTML and Bootstrap and were designed to be user-friendly to researchers (Fig. 1). Moreover, useful bioinformatics tools (including JBrowse, BLAST, and others) are also bundled with genomic data sets in the database for researchers.

**Table 2:** Statistics of the whole data set in the Portal of Juglandaceae (PJU)

Data type	Count
Nuclear genome	9
Choroplastid genome	13
Coding sequence	488,299
Protein	488,299
GO term	1,792,549
KEGG	57,592
miRNA	750
Transcription factor	30,927
Article	31

## Utilization of the PJU

### The portal’s homepage

**Fig. 2:**
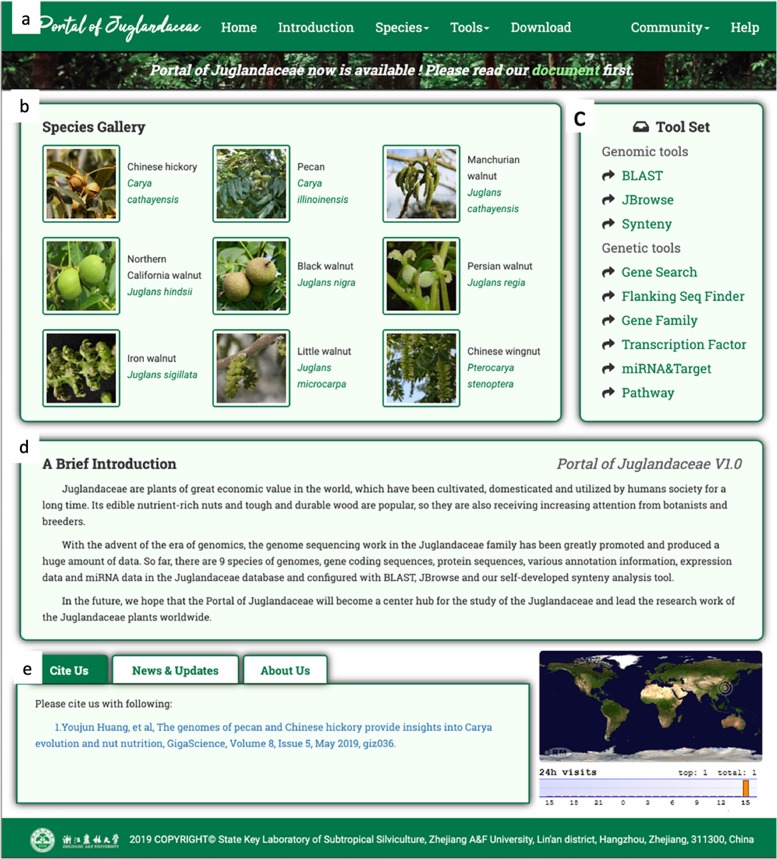
Homepage of the Portal of Juglandaceae (PJU). The PJU homepage contains five components: **a** navigation bar, **b** species gallery, **c** tool set, **d** brief introduction, and **e** news, citation and visitors track. Visitors can browse for desired information using components

A clear and completely displayed homepage of the PJU (Fig. 2) has been built. Currently, the homepage includes five main parts: navigation bars, species galleries, tool sets, brief introductions, and other modules. The navigation bar (Fig. 2a), at the top of the homepage, consists of seven labels: Home, Introduction, Species, Tools, Download, Community, and Help. Under the navigation bar is the species gallery (Fig. 2b) of the PJU showcasing nine species. A tool set (Fig. 2c) containing BLAST, JBrowse, and several other practical search tools is on the right side of the species gallery. Below the species gallery and tool set, a brief introduction (Fig. 2d) of the PJU is given. At the same time, other useful information, including news, citations, and global visitors, is displayed at the bottom of the homepage (Fig. 2e).

### A brief introduction to the Juglandaceae family

In the four parts of the Introduction module, we educate visitors about the Juglandaceae family. The first part describes the origin of the Juglandaceae family. We investigated paleontological and phylogenetic studies on the Juglandaceae family and summarized them in detail in the web pages^30–32^. The second part is for taxonomy. We constructed a tree of the taxonomic status and position of Juglandaceae within the angiosperms and present important economic species of the Juglandaceae family. In the third part, we detail the economic value of the nine existing species in the Juglandaceae database. The fourth section lists the published literature on the Juglandaceae genome study and provides links to the full-text downloads.

In the Species module, we give basic introductions to the nine sequenced species and statistics on genome sequencing and provide users with links to BLAST, JBrowse, searches, and downloads.

### The PJU search kits

**Fig. 3:**
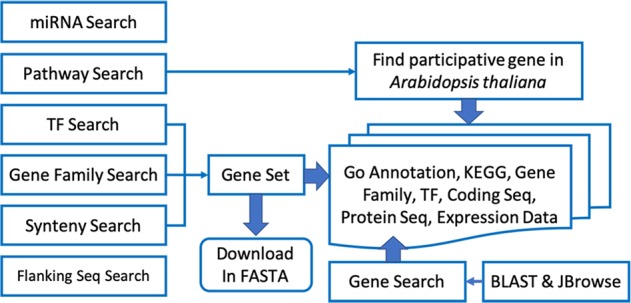
The Portal of Juglandaceae searching flowchart. Scientists can use BLAST and JBrowse to find the homologous gene and its position and then use Gene Search to obtain related information. In addition, scientists can also utilize six search functions to obtain the desired information (left diagram). The DNA or protein sequences of the query gene set in FASTA format are also available for download

We have developed a range of search tools through genomic data mining, including gene, transcription factor, gene family, synteny, miRNA, and pathway searches. A full map of search tools is presented in Fig. 3.

**Fig. 4:**
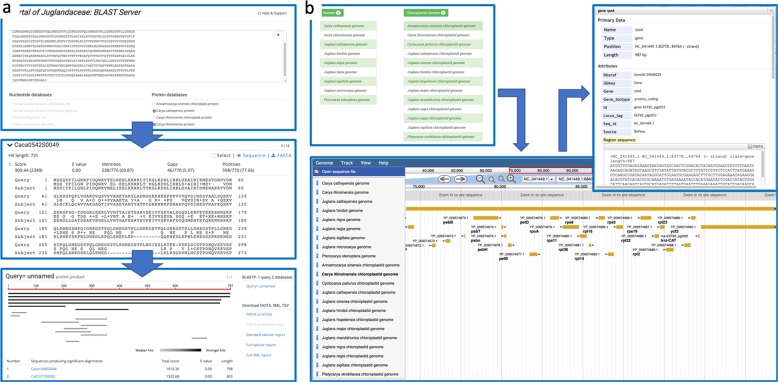
BLAST and JBrowse functions. Scientists can search for Juglandaceae homologous genes by using **a** the BLAST function. Scientists are also able to visualize the location of the query gene in the genome with the help of **b** JBrowse

**Fig. 5:**
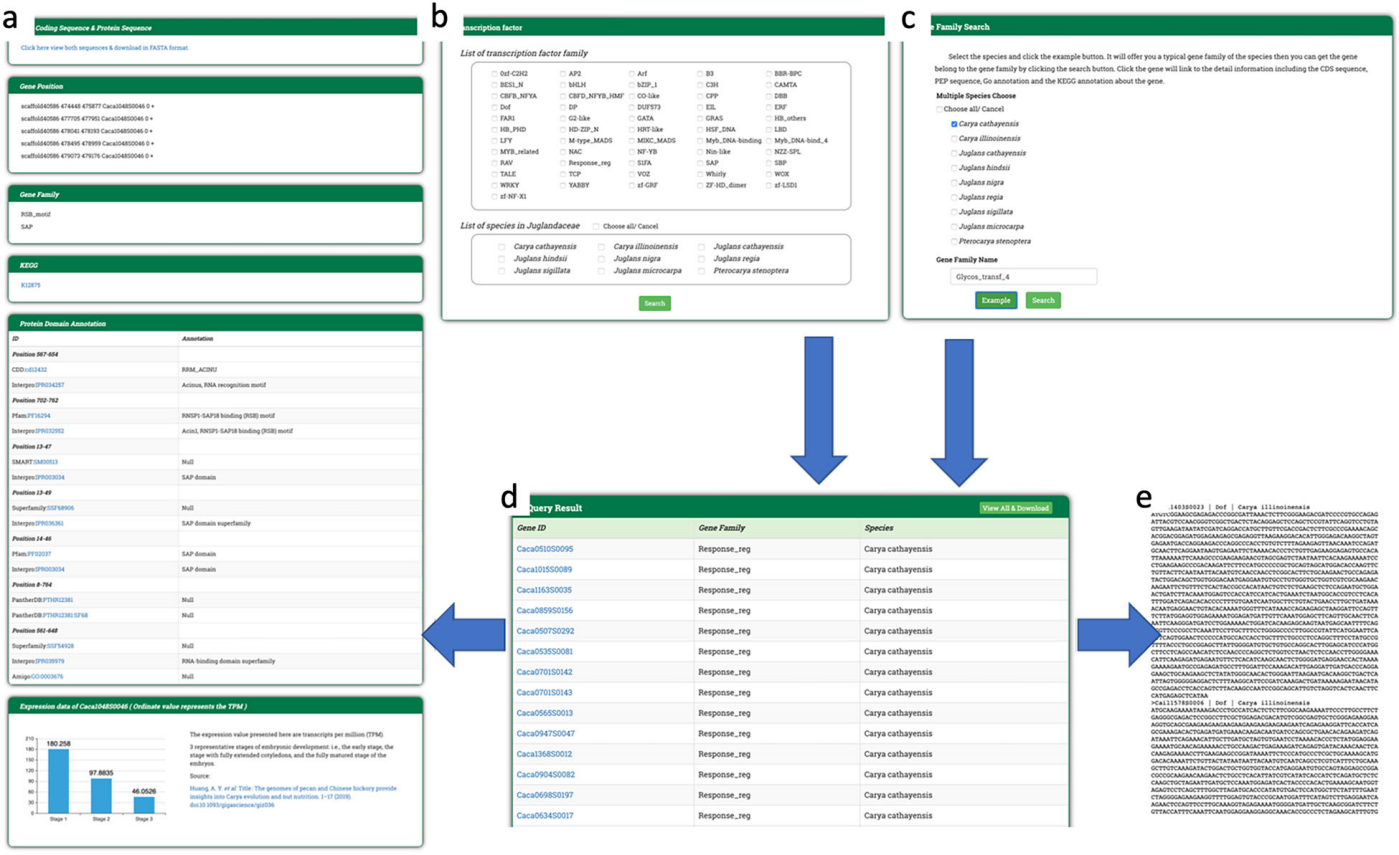
Set of search tools. The Portal of Juglandaceae (PJU) has a set of search tools that help scientists look for desired information. Scientists can enter the desired gene identifier, which is obtained from BLAST search results, to view **a** a report that contains the gene family, KEGG and GO annotations, nucleotide and protein sequences, and gene expression data based on TPM values. Scientists can search against **b** the gene family or **c** transcription factor database by entering a specific gene family or transcription factor name to search for related information. The PJU will return **d** a gene list that links to both **e** the FASTA file for viewing/downloading and detailed information, as mentioned above

On the PJU, scientists can find homologous genes or a desired gene in the Juglandaceae family by utilizing the BLAST tool (Fig. 4a). Scientists can also find the specific location of the desired gene in the genome with the help of JBrowse (Fig. 4b). From the search results of BLAST and JBrowse, scientists can enter the gene’s identifier to search for information about the gene family, KEGG annotation, GO annotation (with links to the corresponding annotation database provided for more information), nucleotide coding and PEPs (FASTA format), and gene expression data based on TPM (Fig. 5).

Additionally, we also provide a feature to search for flanking sequences using the Flanking Sequence Finder module. With this tool, scientists can easily identify key regulatory elements and clone promoters.

The gene family search (Fig. 5b) and transcription factor search (Fig. 5c) functions are all based on specific gene search functions. The scientist selects the species and enters the transcription factor family or gene family name. After the search is conducted, the database returns a list of corresponding gene collections to the user (Fig. 5d). Scientists can click the gene ID, which is linked to detailed information (Fig. 5a) and the FASTA file (Fig. 5e).

**Fig. 6:**
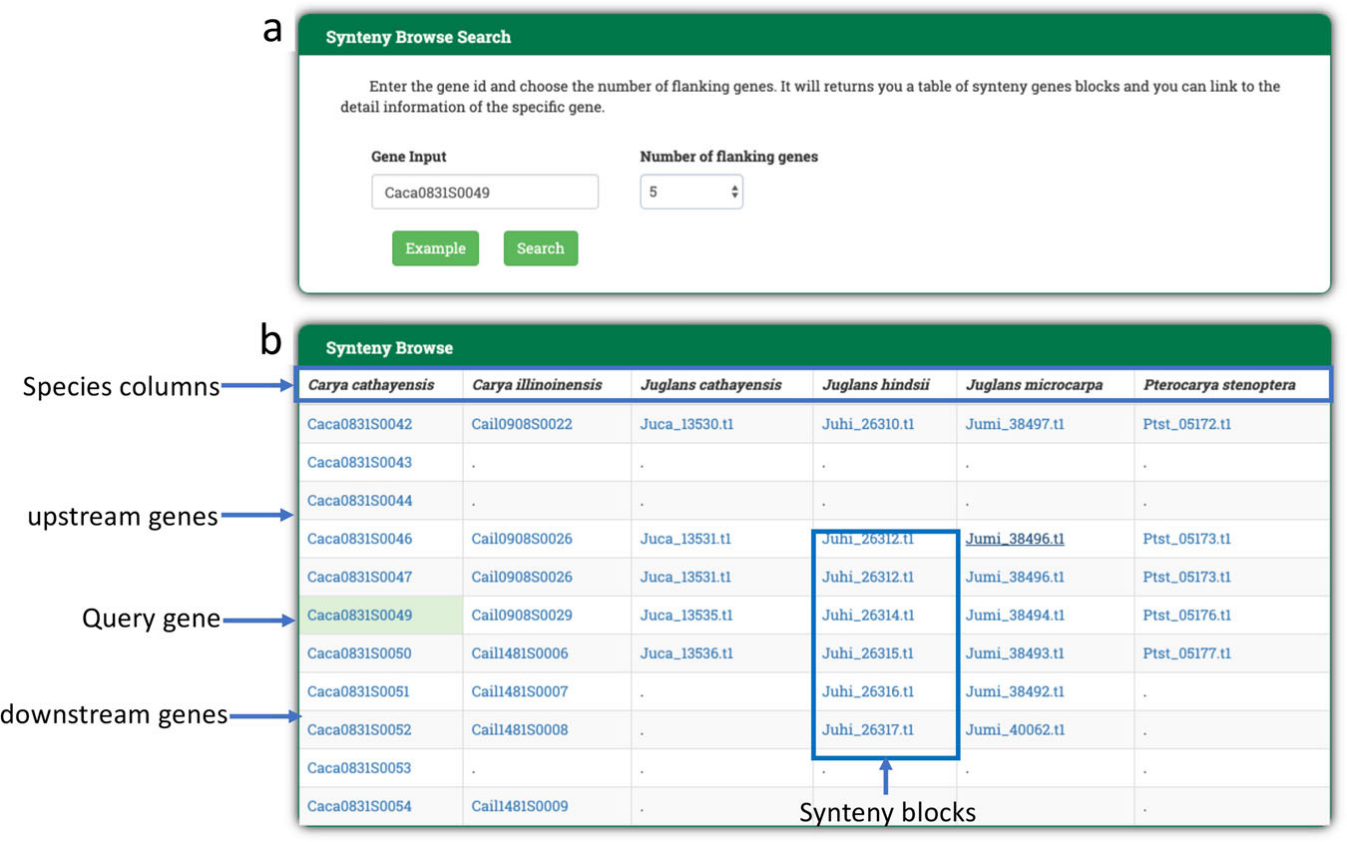
Synteny search function in Portal of Juglandaceae (PJU). Scientists can look for the syntenic genes of Juglandaceae by **a** entering a gene identifier and deciding the number of flanking genes to be presented in the Synteny Browse Search. The PJU will return **b** a table of syntenic gene blocks between Juglandaceae species to scientists

The functionality of the syntenic gene block search is based on Mcscan^27^ software. We searched and organized the syntenic gene blocks for the whole genomes of multiple Juglandaceae species. Scientists can enter the gene ID and flanking gene numbers to view the collinear portion of the gene block against other species’ genomes (Fig. 6).

In the pathway module, we selected important metabolic pathways from the KEGG database and identified some of the key genes in *Arabidopsis thaliana*. Scientists can find the sequences in TAIR^33^ http://arabidopsis.org via the link and BLAST the sequences in our database.

Finally, we also provide miRNA data for two species (*C. cathayensis* and *C. illinoinensis*), and the corresponding precursor sequences in the Juglandaceae family can be found by inputting the miRNA family name.

### Juglandaceae breeding

For the breeding module, we used the existing germplasm repository of our campus to build a phenotype resource library. We focused on the observation and classification of the morphological changes in the pecan inflorescence, pollen vigor, and stigma receptivity between different cultivars. Additionally, we measured several important traits related to nut morphology and fatty acid components.

Although pecan (*C. illinoinensis*) is a monecious tree with diecious flowers, most of its varieties are actually expressed as diecious, which means that they can avoid self-pollination, thus improving the quality, yield, and genetic diversity of the nut^34^. Our research team conducted continual germplasm surveys focusing on flowering and nut characteristics to understand the flower phenological and flower bud differentiation mechanisms.

The phenotypes of wild and domesticated germplasm resources of *C. cathayensis* are still being sampled and collated. We will share these data with the community once they are ready.

### Download and community modules

The Download module provides the genome assembly, CDS and PEPs, annotation data, and miRNA downloads in FASTA and GFF3 formats. Researchers can also directly access data from the chloroplast genome. Data on the expression levels of different genes during three developmental stages^17^ (the early and fully extended stages of cotyledon development and the fully mature stage of embryos) of plant growth and development are presented in CSV format.

For the Community module, we collected the literature on Juglandaceae research. Scientists can search this literature by date, species, or research type.

## Conclusion and future developments

With the rapid development of sequencing technology and corresponding bioinformatics software, an increasing number of plants in the Juglandaceae family have entered the era of research with high-throughput data. How to effectively use and mine this valuable data on a benchtop sequencer will become the focus of modern biological research. We conducted genomic data mining and established the PJU. The PJU aims to be not only a genomic information database but also a user-friendly portal that integrates several useful genomic datasets under a simple website framework.

When new higher quality genomic data emerge, we will collect and analyze them and immediately release them into the PJU. We hope that the Portal of Juglandaceae will be a hub for future genomic research on the Juglandaceae family.
